# Endoplasmic reticulum stress and the unfolded protein response in lung diseases: molecular pathways and therapeutic interventions

**DOI:** 10.1002/path.70058

**Published:** 2026-04-14

**Authors:** Lanlan Song, Yichen Liu, Chen Xu, Yaochen Zhang, Kaiming Xu, Dan Yao, Xiaoying Huang

**Affiliations:** ^1^ Division of Pulmonary Medicine, The First Affiliated Hospital, Wenzhou Medical University, Wenzhou Key Laboratory of Interdiscipline and Translational Medicine Wenzhou Key Laboratory of Heart and Lung Wenzhou PR China; ^2^ Wenzhou Medical University Wenzhou PR China

**Keywords:** endoplasmic reticulum, endoplasmic reticulum stress, unfolded protein response, lung diseases, molecular mechanisms, therapeutic targeting

## Abstract

Endoplasmic reticulum stress (ERS) occurs when the protein‐folding capacity of the endoplasmic reticulum (ER) is overwhelmed, triggering the unfolded protein response (UPR) to restore homeostasis. However, severe or persistent ERS can shift the UPR toward pro‐inflammatory, apoptotic, and fibrotic signaling, thereby exacerbating tissue injury. The pathogenesis and progression of lung diseases, which involve highly heterogeneous cell populations, are significantly influenced by these mechanisms. Indeed, ERS and UPR activation are now recognized as central players in the pathophysiology of numerous lung diseases. This review examines the impact of dysregulated ERS/UPR signaling across different lung diseases, with a particular focus on its cell‐type‐specific effects and disease‐specific implications. Furthermore, we discuss emerging therapeutic strategies designed to modulate these pathways. A comprehensive understanding of the cell‐type‐specific outcomes of ERS/UPR is therefore crucial for developing targeted interventions to mitigate or reverse lung disease progression. © 2026 The Author(s). *The Journal of Pathology* published by John Wiley & Sons Ltd on behalf of The Pathological Society of Great Britain and Ireland.

## Introduction

**Figure 1:**
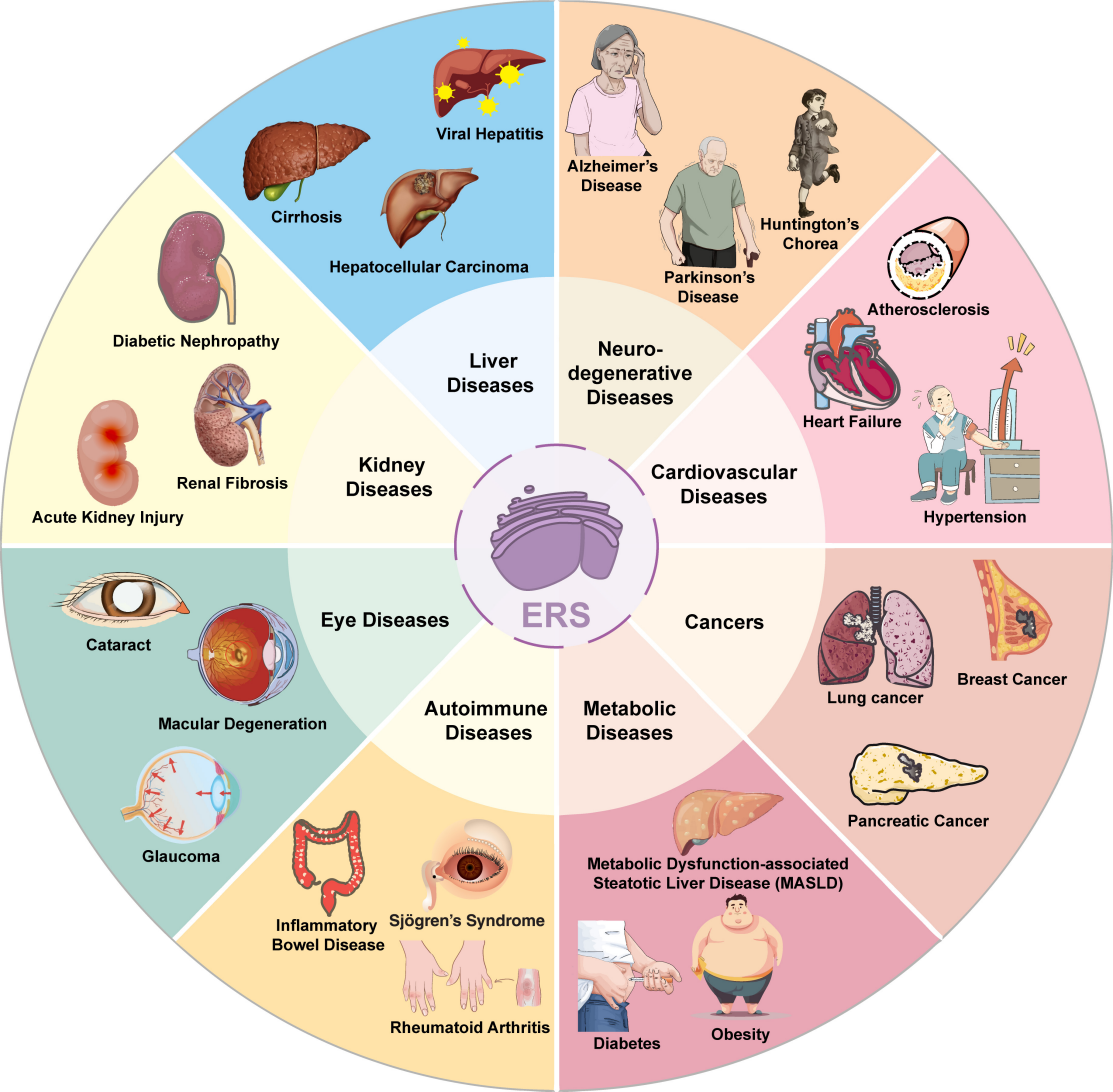
Association of ERS with human diseases. ERS and the UPR are mechanistically linked to the pathogenesis of various diseases, including neurodegenerative diseases, cardiovascular diseases, cancers, metabolic disorders, autoimmune conditions, as well as kidney, liver, and eye diseases.

Endoplasmic reticulum stress (ERS) constitutes a pivotal cellular defense mechanism that is initiated when the protein‐folding capacity of the endoplasmic reticulum (ER) is exceeded by various physiological and pathological insults – such as oxidative stress, calcium dysregulation, or nutrient deprivation. Upon accumulation of unfolded or misfolded proteins beyond a critical threshold, the unfolded protein response (UPR) is triggered. This adaptive signaling cascade is mediated by three key transmembrane sensors: activating transcription factor 6 (ATF6), eukaryotic translation initiation factor 2‐alpha kinase 3 (EIF2AK3), and serine/threonine‐protein kinase/endoribonuclease inositol‐requiring enzyme 1 (IRE1), which collectively orchestrate efforts to restore ER proteostasis. Nevertheless, under prolonged or intense ERS conditions, the UPR shifts from a pro‐survival to a pro‐death program, driving the activation of inflammatory, apoptotic, and fibrotic pathways that ultimately aggravate tissue injury. ERS has been implicated in the pathogenesis of a broad spectrum of human diseases, including Alzheimer's disease, Parkinson's disease, diabetes, and cancer (Figure 1).

**Figure 2:**
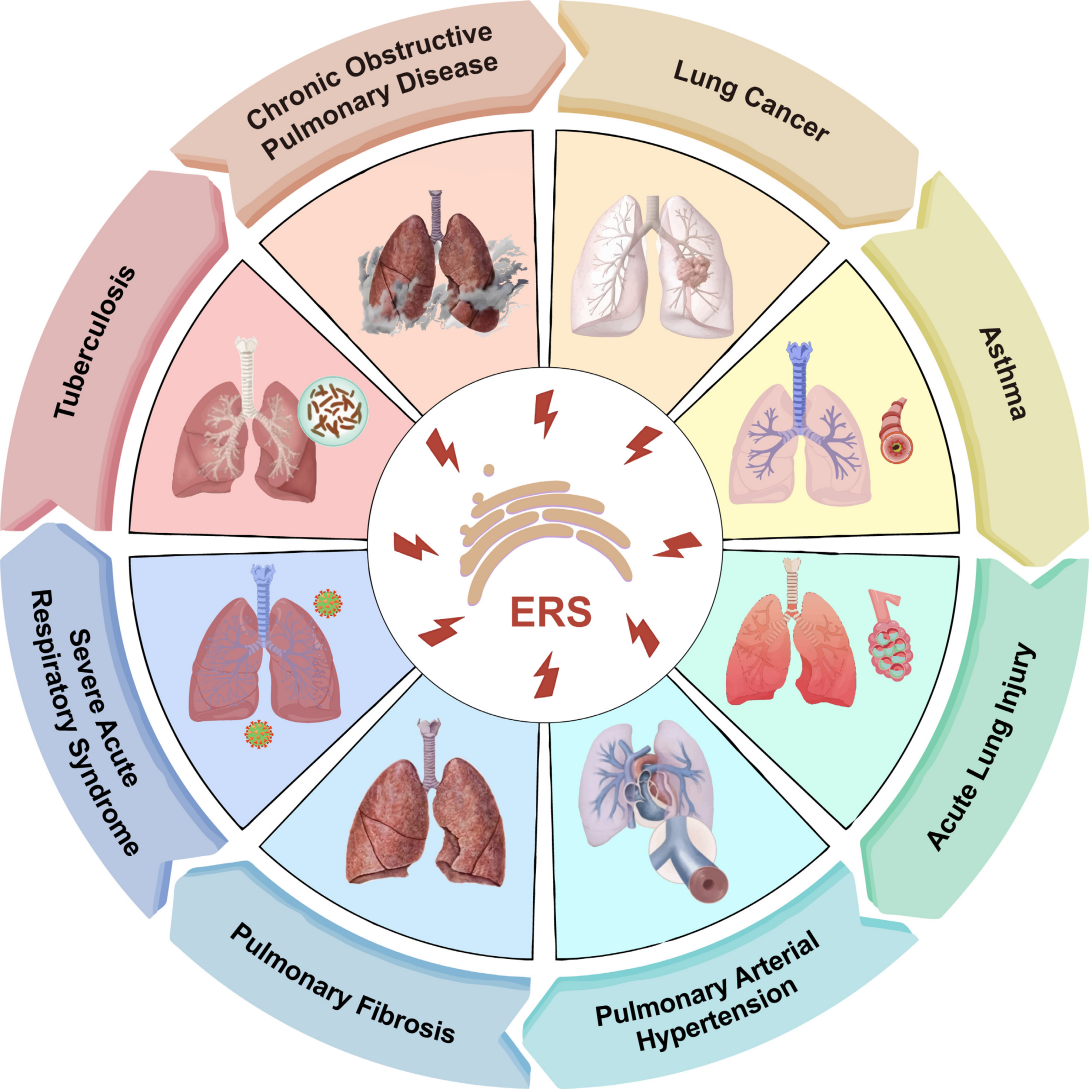
Association of ERS with various lung diseases. This diagram highlights the role of ERS in a spectrum of lung diseases.

The lungs are a complex organ composed of a multitude of different cell types, each contributing uniquely to pulmonary function and disease pathology. In the context of lung diseases, ERS has emerged as a critical factor in pathogenesis, triggering cell‐type‐specific responses that drive distinct outcomes in processes of repair, pathogenesis, and homeostasis restoration following injury (Figure 2). This review focuses on the molecular mechanisms of ERS and the UPR, describing their roles in cell‐specific UPR mechanisms within lung diseases. It also explores the therapeutic potential of targeting these pathways for precision medicine in lung diseases.

## Molecular mechanisms of ERS and the UPR in cellular fate regulation

ERS occurs when protein‐folding demand exceeds the ER's capacity, leading to the accumulation of misfolded proteins. Triggered by stressors such as hypoxia, oxidative stress, or nutrient deprivation, ERS disrupts ER homeostasis and activates the UPR, which initially restores proteostasis. The UPR orchestrates a sophisticated adaptive response by initiating transcriptional reprogramming to alleviate the protein load within the endoplasmic reticulum, attenuating translation to curb new protein synthesis, enhancing the endoplasmic reticulum‐associated degradation (ERAD) of misfolded proteins, and promoting autophagy‐mediated clearance. If these mechanisms fail to restore homeostasis, the UPR initiates apoptosis to eliminate irreparably damaged cells.

**Figure 3:**
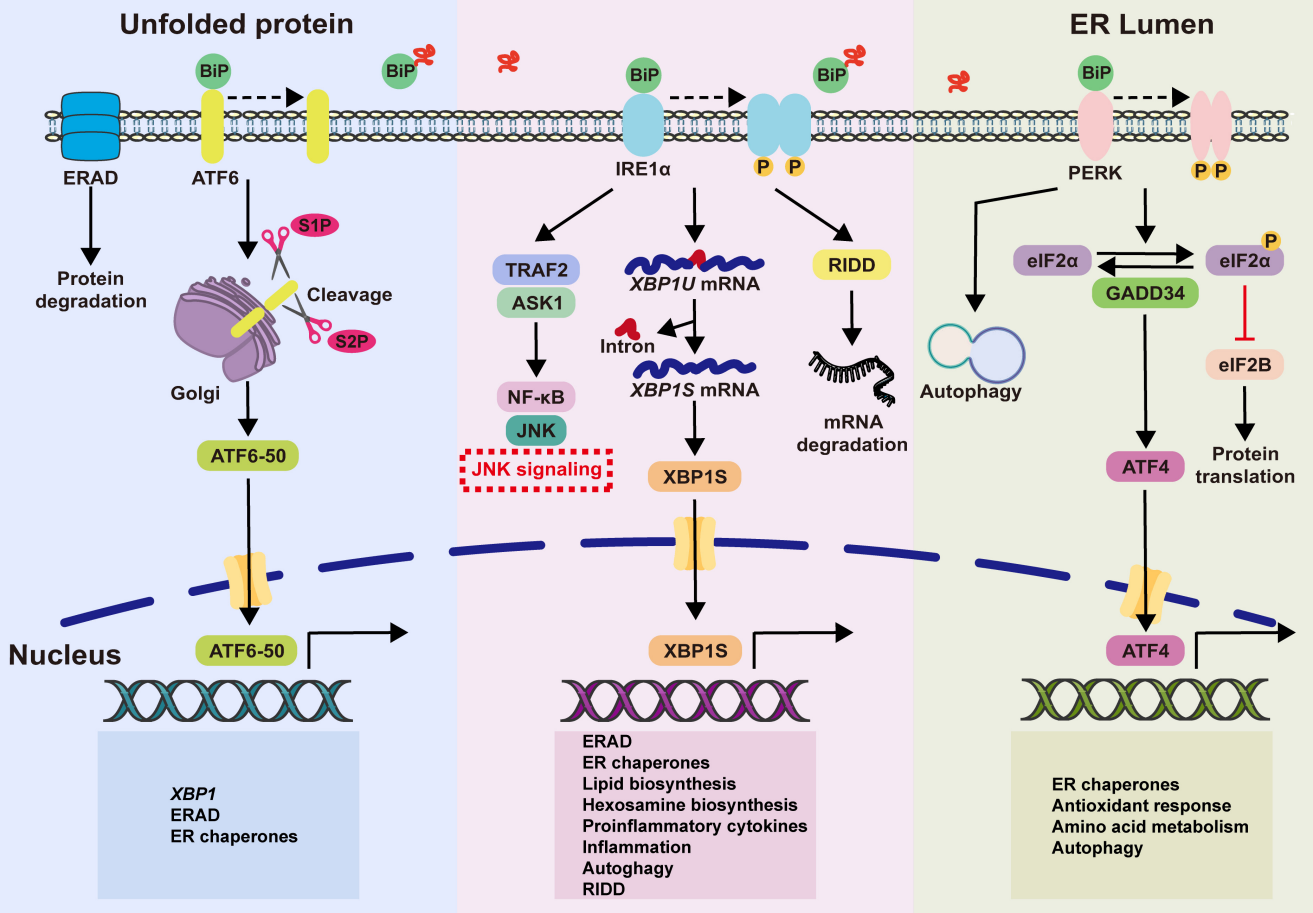
Core signaling pathways of the UPR. Under ERS conditions, the IRE1–XBP1, EIF2AK3–eIF2A, and ATF6 pathways are coordinately activated to mitigate proteotoxic stress.

The UPR is mediated by three ER‐resident transmembrane sensors: IRE1, EIF2AK3, and ATF6. Under basal conditions, these sensors are maintained in an inactive state by binding to the ER chaperone binding immunoglobulin protein (BiP)/endoplasmic reticulum chaperone BiP (HSPA5). During ERS, BiP/HSPA5 dissociation triggers their activation, initiating three parallel signaling cascades that collectively determine cellular fate (Figure 3).

## UPR signaling pathways

### IRE1–XBP1 pathway

IRE1, a type I transmembrane protein containing luminal stress‐sensing domains and cytoplasmic kinase/RNase domains, exists as inactive monomers bound to BiP/HSPA5 under homeostasis. ERS‐induced BiP/HSPA5 dissociation promotes IRE1 homodimerization and autophosphorylation, activating its RNase domain. This RNase activity catalyzes unconventional splicing of X‐box binding protein 1 (*XBP1*) mRNA, removing a 26‐nucleotide intron to generate the potent transcriptional activator spliced X‐box binding protein 1 (XBP1s). XBP1s upregulates genes involved in ERAD, lipid biosynthesis, and chaperone expression. Additionally, IRE1 mediates two key processes: (1) regulated IRE1‐dependent decay (RIDD) of specific mRNAs; and (2) TNF receptor‐associated factor 2 (TRAF2)‐dependent activation of the mitogen‐activated protein kinase 5 (MAP3K5)‐stress‐activated protein kinase (JNK)/p38 mitogen‐activated protein kinase (MAPK) cascade.

### EIF2AK3–EIF2S1 pathway

EIF2AK3 (also known as PERK), a Ser/Thr kinase, undergoes dimerization and autophosphorylation during ERS in a BiP/HSPA5 dissociation‐dependent manner. Activated EIF2AK3 phosphorylates eukaryotic initiation factor 2A (eIF2A) at Ser51, resulting in (1) global translational attenuation via eukaryotic initiation factor 2B (eIF2B) inhibition and (2) preferential translation of activating transcription factor 4 (*ATF4*) mRNA through upstream open reading frame (ORF) bypass. *ATF4* initiates a biphasic transcriptional response: early induction of cytoprotective genes, followed by DNA damage‐inducible transcript 3 protein (DDIT‐3)‐mediated apoptosis through B‐cell lymphoma 2 (BCL‐2) suppression during prolonged stress.

### ATF6 proteolytic activation pathway

ATF6, a type II transmembrane protein, exists in BiP/HSPA5‐bound oligomeric states stabilized by disulfide bonds. ERS induces ATF6 monomer release and coat protein complex II (COPII)‐dependent Golgi transport, where sequential cleavage by site‐1 protease (S1P) and site‐2 protease (S2P) generates the transcriptionally active ATF6 fragment (ATF6f, also termed ATF6‐50 or p50‐ATF6). ATF6f translocates to the nucleus to upregulate UPR targets including BiP/HSPA5, endoplasmin (GRP94), and DDIT‐3.

## ER quality control systems in UPR regulation

The ER employs a multi‐tiered quality control network: (1) Chaperone system: BiP/HSPA5 serves as the master regulator, while GRP94 specializes in glycoprotein maturation; (2) Redox machinery: the protein disulfide‐isomerase (PDI)‐ERO1‐like protein alpha/beta (ERO1α/β) system catalyzes disulfide bond formation; however, overactivation leads to reactive oxygen species (ROS) accumulation; (3) Glycoprotein control: the calnexin–calreticulin cycle collaborates with protein disulfide‐isomerase A3 (PDIA3) to monitor *N*‐glycosylated proteins, targeting misfolded substrates for ERAD.

### ERS‐induced apoptosis

**Figure 4:**
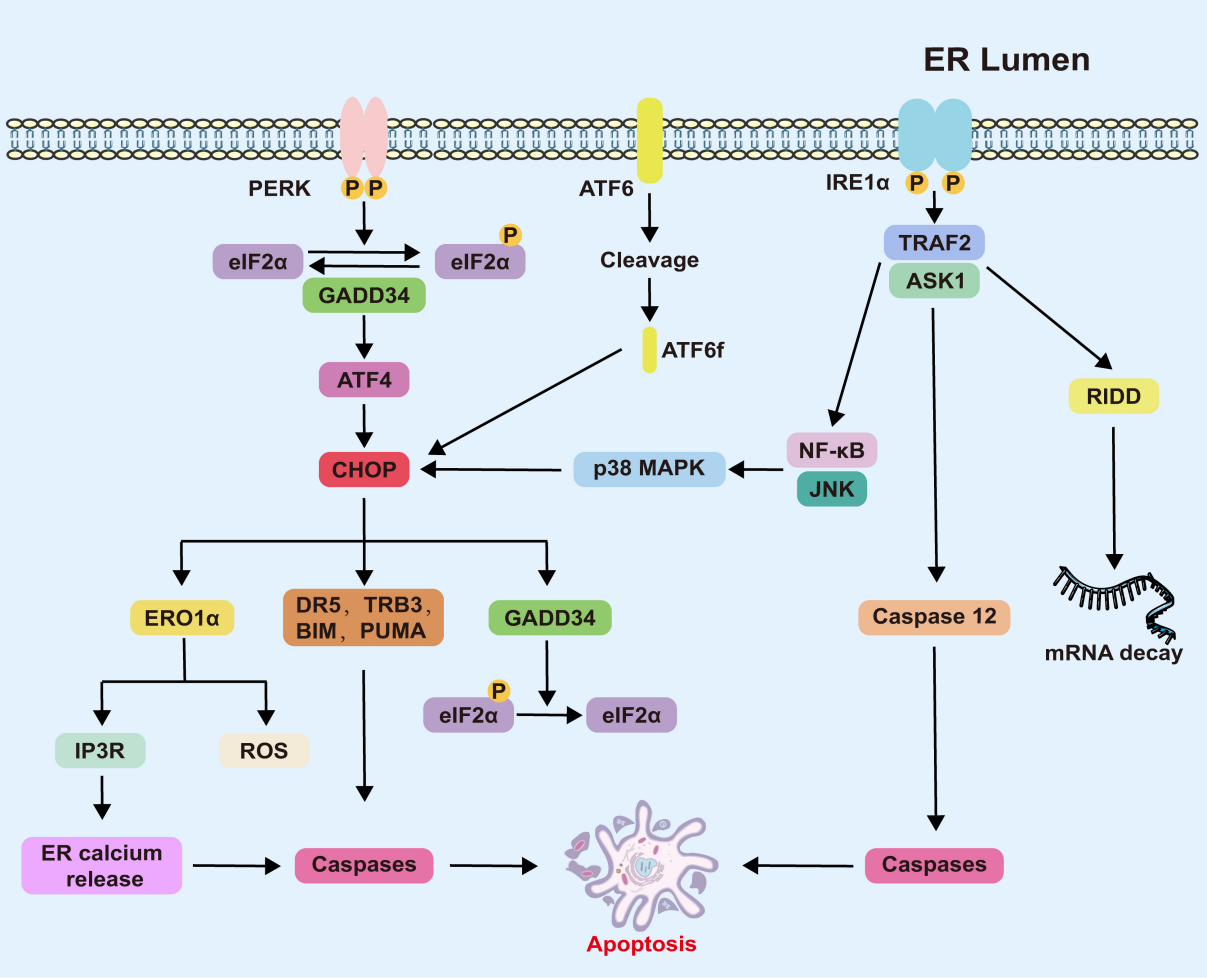
Molecular pathways of ERS‐induced apoptosis. Schematic representation of three convergent apoptotic mechanisms triggered by severe ERS: (1) DDIT‐3‐mediated; (2) caspase‐12‐dependent; and (3) activation of the IRE1–TRAF2–MKK5 axis.

Under severe ERS, cells activate three apoptotic pathways to eliminate irreversibly damaged cells: (1) DDIT‐3‐dependent apoptosis via ERO1α‐mediated ROS production; (2) caspase‐12‐mediated apoptosis through ER‐localized caspase cascade initiation; and (3) IRE1‐triggered apoptosis involving MKK5–JNK–MAPK activation, regulated RIDD of specific mRNAs, and TRAF2–JNK–NF‐kappa‐B inhibitor‐interacting Ras‐like protein (NF‐κB) signaling. As shown in Figure 4, these mechanisms illustrate the shift from adaptive responses to programmed cell death during ERS.

## ERS in lung diseases

Not only is ERS recognized as a pivotal mechanism underpinning multiple lung diseases, but its biomarkers are also closely linked to disease severity and prognosis (supplementary material, Table S1). The lung's complex cellular ecosystem exhibits remarkable heterogeneity in ERS responses, characterized by cell‐type‐specific activation thresholds, temporal dynamics, and functional outcomes (supplementary material, Figure S1). Such compartmentalization of ERS pathophysiology not only shapes patterns of disease susceptibility but also opens avenues for cell‐selective therapeutic interventions. A systematic dissection of these cell‐type‐specific mechanisms will be crucial for advancing precision medicine in pulmonary diseases.

### Chronic obstructive pulmonary disease (COPD)

COPD pathology is sustained by a multicellular network in which epithelial cells, endothelial cells, neutrophils, and alveolar macrophages are among the most prominently implicated players. Cigarette smoke (CS) remains the dominant environmental trigger of ERS, generating oxidants and electrophiles that disturb ER proteostasis across resident lung cells. Rather than inducing a uniform response, CS elicits cell‐type‐specific UPR signatures that collectively shape the heterogeneous lesions characteristic of COPD.

#### Epithelial cells

ERS plays a pivotal role in COPD pathogenesis by disrupting bronchial and alveolar epithelial homeostasis through multiple interconnected mechanisms. CS‐induced oxidation of PDI compromises protein‐folding capacity, triggering UPR activation and subsequent apoptosis [1]. ERS exhibits dual regulation of autophagy, with the EIF2AK3–eIF2A axis promoting cell survival through cytoprotective autophagy, while the DDIT‐3–*ATF‐4* pathway induces detrimental ER‐phagy and apoptosis [2]. Furthermore, ERS amplifies oxidative stress via BiP/DDIT‐3‐dependent ROS production and stimulates NF‐κB‐mediated inflammation through the BiP–*ATF‐4*–DDIT‐3 signaling axis, creating a self‐perpetuating cycle of epithelial damage [3]. ERS also drives epithelial–mesenchymal transition (EMT) through the IRE1/SMAD family member 2/3 (SMAD2/3) signaling axis, contributing to airway remodeling and dysfunction [4]. At the mitochondrial level, ERS triggers mitofusin‐2‐dependent hyperfusion, an adaptive response that paradoxically enhances cellular vulnerability to additional stressors [5].

#### Endothelial cells

ERS exhibits dual regulation of endothelial cell fate in response to CS exposure. In naive endothelium, ERS induces apoptotic cell death through suppression of eIF2A phosphorylation and focal adhesion kinase 1 (FADK 1) signaling pathways, coupled with impaired UPR, thereby promoting strain‐specific emphysema development [6]. Conversely, chronic smoke exposure drives an adaptive shift to apoptosis resistance in microvascular endothelium, where ERS‐mediated AKT serine/threonine kinase (AKT) activation redirects ceramide signaling from pro‐apoptotic to pro‐autophagic pathways, maintaining cell survival despite persistent ERS [7].

#### Neutrophils

Neutrophilic inflammation is prevalent in the airways of COPD patients. In COPD, neutrophils develop a pathological phenotype featuring impaired bactericidal activity but exaggerated degranulation, resulting in excessive neutrophil elastase release and subsequent tissue injury. The IRE1–XBP1s axis sustains this pathological secretory phenotype, implicating IRE1 inhibitors as potential therapeutics for attenuating elastase‐driven tissue injury [8, 9].

#### Macrophages

Alveolar macrophages (AMs) play a pivotal role in CS‐induced lung injury through ERS‐mediated mechanisms. CS exposure activates AMs, triggering oxidative stress, upregulation of ERS markers, and excessive pro‐inflammatory cytokine production, thereby exacerbating pulmonary damage [10]. ERS exerts dual regulation on macrophage polarization, sustaining the pro‐inflammatory, classically activated macrophage phenotype (M1) through the nuclear respiratory factor 1 (NRF1)/nuclear factor erythroid 2‐related factor 2 (NRF2)–sequestosome‐1 (SQSTM1/p62) signaling axis, while promoting the alternative anti‐inflammatory macrophage phenotype (M2) via interferon regulatory factor 4 (IRF4) upregulation, collectively contributing to COPD pathogenesis [11]. Furthermore, ERS critically impairs macrophage efferocytic capacity through EIF2AK3/EIF2S1 (eIF2A)‐dependent activation of the Ras homolog family member A (RhoA)–Rho‐associated protein kinase (ROCK) pathway, representing a fundamental mechanism underlying CS‐induced phagocytic dysfunction in chronic lung diseases [12].

### Lung cancer

Lung cancer comprises several histological subtypes whose initiation, progression, and resistance to therapy are increasingly tied to ERS. Tobacco carcinogens, hypoxia, nutrient deprivation, and oncogenic mutations disturb ER proteostasis and activate the UPR. The UPR then exerts distinct effects in different cell types, shaping the course of the disease.

#### Cancer epithelial cells

The UPR in lung cancer epithelial cells demonstrates a dual, context‐dependent role that significantly impacts tumor progression and treatment responses. Under moderate stress conditions, the UPR exhibits pro‐tumorigenic effects through upregulation of BiP/HSPA5, which enhances ERS adaptation and facilitates oncogenesis [13, 14]. Concurrently, UPR‐induced metabolic reprogramming supports tumor cell proliferation via glycolytic activation [15], while its induction of EMT contributes to therapy resistance [16]. In contrast, under severe ERS, the UPR exerts tumor‐suppressive effects through distinct mechanisms: autophagy [17], apoptosis [18, 19], immunogenic cell death [20], ROS/mitochondrial dysfunction [21, 22], and non‐canonical death pathways such as ferroptosis [23] and paraptosis [24]. The UPR can also inhibit tumor growth by modulating the cell cycle [25] and inducing cellular senescence [26].

#### Myeloid‐derived suppressor cells (MDSCs)

Within the myeloid compartment, ERS exhibits dual immunomodulatory effects, balancing immunosuppressive and immunostimulatory responses. On the one hand, it promotes immunosuppression through dysregulation of tumor necrosis factor (TNF)‐related apoptosis‐inducing ligand receptor (TRAIL‐R) in MDSCs [27] and on the other hand, it confers resistance to programmed cell death protein 1 (PD‐1) blockade via ERO1α overexpression [28].

#### Dendritic cells (DCs)

ERS induces immunogenic calreticulin exposure that activates DCs through cluster of differentiation 91 (CD91)‐mediated antigen presentation, while simultaneously synergizing with cluster of differentiation 47 (CD47) blockade to enhance phagocytic efficiency and T‐cell priming [29, 30]. Importantly, this EIF2AK3‐mediated stress response further orchestrates dendritic cell infiltration and activation within the tumor microenvironment, where their interaction with immune markers ultimately influences cancer prognosis [31].

#### Lymphoid cells

ERS exhibits complex immunomodulatory effects on lymphoid cells, with both pro‐ and anti‐tumor immunity consequences. On the immune‐activating side, ERS enhances natural killer (NK) cell cytotoxicity through upregulation of ecto‐calreticulin and retinoic acid early transcript 1 (RAE1) ligands [32], while also promoting CD8^+^ T‐cell tumor infiltration [29] and enhancing their effector function in NSCLC through ROS‐dependent immunogenic modulation and calcium‐mediated apoptotic signaling [33]. However, ERS simultaneously drives immunosuppressive mechanisms, including GRP94‐dependent regulatory T‐cell (Treg) expansion [34] and suppression of interferon‐gamma (IFN‐γ) production in T helper 1 (Th1) cells, which impairs CD8^+^ T cell‐mediated anti‐tumor responses in lung squamous cell carcinoma [35]. Intriguingly, the XBP1 branch of ERS signaling exhibits paradoxical immunostimulatory effects by modulating cholesterol metabolism to reduce Treg suppression and alleviate CD8^+^ T‐cell exhaustion [36]. Additionally, the ERS effector reticulon‐4 isoform B (RTN4‐B) selectively inhibits T helper 2 (Th2) cell responses [37], further illustrating the complex, multifaceted nature of ERS‐mediated immunoregulation in the tumor microenvironment.

### Asthma

Allergic asthma is characterized by Th2 cell, mast cell, and eosinophil infiltration, along with airway remodeling such as goblet cell metaplasia and basement membrane thickening. Common triggers include inhaled allergens, often combined with respiratory infections or air pollutants such as diesel exhaust, cigarette smoke, and ozone. ERS modulates the function of various airway‐resident cells, contributing to chronic inflammation and remodeling in asthma.

#### Bronchial epithelial cells

ERS plays a pivotal role in asthma pathogenesis by orchestrating airway epithelial dysfunction through distinct yet interconnected mechanisms. In fungal‐sensitized asthma, ERS synergizes with airway epithelial PI3K‐δ to amplify allergic inflammation [38]. The ATF4–ATF6–XBP1 axis drives EMT [39], while simultaneously DDIT‐3‐mediated apoptosis contributes to epithelial barrier breakdown [40]. These processes are further exacerbated by ERS‐dependent epithelial fibrosis [41]. At the cellular level, ERS triggers pyroptosis through activation of the cyclic GMP‐AMP synthase (cGAS)–stimulator of interferon genes protein (STING)–ERS pathway [42], amplifying inflammatory responses. Chronic ERS disrupts the differentiation and repair functions of airway epithelial stem/progenitor cells [43]. This impairment, coupled with direct activation of goblet cells, leads to pathologic mucus hypersecretion dominated by mucin 5AC (MUC5AC) overproduction [44].

#### Airway smooth muscle cells

ERS triggers asthma‐associated airway smooth muscle dysfunction through selective activation of the ATF6 pathway, which induces calcium dysregulation and upregulates remodeling factors, ultimately leading to hypertrophy, fibrosis, and hyperresponsiveness [45, 46].

#### Macrophages

ERS critically drives pathogenic M2 polarization in asthma by suppressing anti‐inflammatory interleukin 10 (IL‐10) and PD‐1 signaling while activating a DDIT‐3‐dependent interleukin 4 (IL‐4)/signal transducer and activator of transcription 6 (STAT6)/transcription factor EC (TFEC)/IL‐4 receptor subunit alpha (IL‐4Rα) positive feedback loop. Genetic deletion of DDIT‐3 attenuates airway hyperresponsiveness and remodeling, confirming ERS as a key regulator of macrophage‐mediated asthma pathogenesis [47, 48].

#### Eosinophils and neutrophils

ERS differentially regulates asthma‐associated inflammation: in eosinophilic asthma, the IRE1–XBP1 axis drives eosinophil activation and survival [49], while in neutrophilic asthma, EIF2AK3/DDIT‐3‐mediated ERS promotes neutrophil differentiation [8, 50]. Concurrently, IRE1 enhances T helper 17 (Th17) responses, exacerbating neutrophilia [51]. These UPR‐activated pathways collectively amplify Th2/Th17 cytokines, inflammasome activity, and apoptosis, linking proteostatic stress to inflammatory airway dysfunction [52].

### Acute lung injury (ALI)

ALI is a severe inflammatory condition caused by sepsis, mechanical ventilation, or environmental exposures, marked by breakdown of pulmonary endothelial–epithelial barriers. ERS drives cell‐type‐specific pathogenic responses in lung parenchyma, critically influencing disease progression.

#### Epithelial cells

In alveolar epithelium, mechanical ventilation triggers EIF2AK3‐mediated ERS that exacerbates inflammation and barrier dysfunction [53]. In ALI, ERS promotes pathogenic responses by stimulating exosomal release from epithelial cells, which amplifies inflammation through Toll‐like receptor 4 (TLR4)/NF‐κB‐mediated macrophage pyroptosis [54]. Under hyperoxic conditions, ERS additionally triggers the release of caspase‐3‐positive extracellular vesicles (EVs) from epithelial cells, activating AMs and further exacerbating inflammation [55]. ERS also orchestrates distinct cell death programs – EIF2AK3/ATF4 signaling mediates LPS‐induced autophagic cell death [56], while EIF2AK3‐dependent mitochondrial dysfunction through ROS overproduction drives ferroptosis [57]. Additionally, the IRE1–XBP1 axis contributes to maladaptive repair by activating β‐catenin signaling in injured epithelium [58].

#### Endothelial cells

ERS critically impairs endothelial function through multiple mechanisms. By suppressing stress‐associated endoplasmic reticulum protein 1 (SERP1), ERS triggers endothelial apoptosis and disrupts vascular integrity [59], while simultaneously activating inflammatory pathways through BiP‐regulated NF‐κB signaling [60]. The stress response promotes vascular leakage in acute lung injury via mechanistic target of rapamycin (mTOR)‐dependent oxidative stress [61] and mediates seawater‐induced endothelial damage through dual proliferative arrest and apoptosis [62]. Notably, ERS disrupts vascular homeostasis by dysregulating the angiotensin II (ANGII)–angiotensin‐converting enzyme 2 (ACE‐2)–angiotensin‐(1–7) (ANG1–7) axis [63].

#### Macrophages

ERS drives macrophage dysfunction in acute lung injury through multiple pathways. It triggers TLR4/NF‐κB‐mediated pyroptosis through epithelial exosome release [54] and induces chemokine production in alveolar macrophages upon diesel exhaust exposure [64]. Furthermore, ERS mediates both macrophage apoptosis [65] and ferroptosis [66] via the EIF2AK3/ATF4 signaling pathway during acute lung injury.

#### Neutrophils

ERS plays a pivotal role in neutrophil‐mediated pathogenesis of ALI through multiple mechanisms. ERS exacerbates neutrophil activation by promoting C5a receptor‐triggered granule release through the IRE1–XBP1 pathway [67], while simultaneously potentiating neutrophil extracellular trap (NET) formation via guanine nucleotide‐binding protein G(q) subunit alpha/guanine nucleotide‐binding protein subunit alpha‐11 (GNAQ/GNA11)‐mediated ERS‐dependent neutrophil extracellular trap formation (NETosis) [68]. These ERS‐driven processes lead to enhanced elastase release and pro‐inflammatory cytokine production [69], ultimately contributing to endothelial barrier disruption and pulmonary inflammation [70].

### Pulmonary arterial hypertension (PAH)

PAH is a progressive vascular disorder characterized by elevated pulmonary artery pressure, driven by excessive proliferation of smooth muscle cells. Environmental factors such as hypoxia and viral infections contribute to disease development. Emerging evidence positions ERS as a key driver of PAH pathogenesis, acting through cell‐type‐specific mechanisms in both endothelial and smooth muscle cells.

#### Pulmonary artery smooth muscle cells (PASMCs)

ERS drives PASMC proliferation and vascular remodeling through ATF6 activation, which mediates RTN4‐dependent ER‐mitochondrial uncoupling [71] and lipocalin‐2‐induced iron overload [72]. These ATF6‐dependent changes promote oncogenic metabolic reprogramming, calcium dysregulation, and apoptosis resistance in PASMCs [71, 73]. In addition to the ATF6 branch, the IRE1–XBP1 pathway synergistically promotes PAH progression by XBP1s‐dependent activation of phosphorylated JNK (p‐JNK) and MAPK [74]. Additionally, endothelin‐1 (ET‐1) amplifies ERS by co‐activating ATF6 and XBP1s to enhance pro‐inflammatory responses [75]. Central to these effects is mitochondrial dysfunction, driven by ROS overproduction [76] and exacerbated ER–mitochondrial uncoupling [71, 73]. Paradoxically, while moderate ERS supports PASMC survival, chronic ERS triggers DDIT‐3/caspase‐12‐mediated apoptosis [77], revealing its dual role in PAH pathogenesis.

#### Pulmonary artery endothelial cells (PAECs)

ERS disrupts PAEC function in PAH through a cascade of pathogenic events. Activation of the EIF2AK3–DDIT‐3 pathway induces PAEC apoptosis while disrupting vasoregulatory nitric oxide (NO)/ET‐1 balance [78], creating initial endothelial injury. This injury progresses as sustained ERS triggers phenotypic transformation through endothelial‐to‐mesenchymal transition, endowing PAECs with smooth muscle‐like remodeling properties [79]. Concurrently, ERS disrupts fundamental cellular homeostasis via mitochondrial–ER calcium dysregulation [80], ultimately driving vascular remodeling in PAH.

#### Macrophages

ERS drives macrophage‐mediated vascular inflammation in PAH through mitochondrial dysfunction, ROS overproduction, and p38 MAPK activation, amplifying pathological remodeling [81, 82].

### Pulmonary fibrosis (PF)

In PF, the delicate alveolar architecture is progressively replaced by dense extracellular matrix, persistent myofibroblast activation, and loss of alveolar epithelial cells. This remodeling is initiated by diverse triggers – hereditary surfactant‐processing mutations, inhaled particulate matter, latent viral genomes, chronic hypoxia, age‐related redox shifts, etc. – that converge on saturation of the ER‐folding capacity, with ERS subsequently orchestrating pathology through cell‐type‐specific mechanisms.

#### Alveolar epithelial type II cells (AECII)

ERS critically drives AECII dysfunction in PF through distinct yet interconnected mechanisms. Genetic mutations (*SFTPC*, *SFTPA2*, *ABCA3*) impair protein folding, inducing persistent ERS [83, 84, 85], while hypoxia exacerbates ERS via ER calcium depletion [86]. These insults trigger a cascade of pathological events: DDIT‐3‐mediated apoptosis via caspase‐4/12 activation and mitochondrial dysfunction [87, 88]; EIF2AK3/IRE1‐dependent EMT [89, 90]; and HSPA5 deficiency‐induced senescence and impaired regenerative capacity [43]. ERS establishes feed‐forward loops by impairing mitochondrial function through PINK1 downregulation [88] and inducing pro‐inflammatory responses.

#### Fibroblasts

ERS orchestrates myofibroblast activation in PF through a multifaceted signaling network that converges on pathological ECM deposition. Central to this process is the bidirectional reinforcement between ERS and transforming growth factor beta 1 (TGF‐β1) signaling, where TGF‐β1 upregulates ERS markers while ERS enhances TGF‐β1 responsiveness through thioredoxin domain‐containing protein 5 (TXNDC5)–transforming growth factor beta receptor 1 (TGFBR1) interaction, collectively driving alpha smooth muscle actin (α‐SMA) expression and collagen I overproduction [91, 92, 93]. Beyond TGF‐β1 amplification, ERS sustains fibrogenic activity through parallel mechanisms: IRE1‐mediated autophagy impairment maintains myofibroblast survival [94, 95], while PI3K/AKT activation promotes proliferative persistence [96].

#### Macrophages

ERS critically regulates macrophage function in PF through coordinated molecular pathways. DDIT‐3‐mediated apoptosis is induced via EIF2AK3, ATF6, and IRE1 activation when ROS levels exceed cellular repair capacity [97, 98], while parallel JNK and peroxisome proliferator‐activated receptor gamma (PPARγ) signaling drives macrophage polarization toward an M2 phenotype [99]. These activated macrophages subsequently secrete pro‐fibrotic mediators including TGF‐β, chemokines, and matrix metalloproteases [100, 101], creating a self‐reinforcing cycle of fibroblast activation and myofibroblast transformation that drives progressive PF.

#### Th17 cell

ERS promotes Th17 polarization while suppressing Th1/Th2 differentiation, contributing to fibrosis via interleukin 17 (IL‐17)‐mediated epithelial–fibroblast crosstalk. Environmental stressors such as PM2.5 (particulate matter less than 2.5 μm in diameter) and hypoxia exacerbate this pathway, linking metabolic stress to fibrotic progression [102, 103, 104].

### Severe acute respiratory syndrome coronavirus 2 (SARS‐CoV‐2)

SARS‐CoV‐2 infection imposes a sustained proteotoxic burden on the pulmonary ER. Rather than triggering a terminal UPR, the virus repurposes the UPR to expand replication‐competent membranes, attenuate innate immune signaling, and modulate cytokine production. The resulting cell‐type‐specific reprogramming of airway epithelial cells and myeloid infiltrates underlies the pathophysiological spectrum observed in COVID‐19.

#### Epithelial cells

SARS‐CoV‐2 infection of epithelial cells triggers ERS, which the virus exploits through distinct mechanisms to promote replication and pathogenesis. Viral entry via angiotensin‐converting enzyme 2 (ACE2) receptors leads to ER processing of viral proteins, activating the UPR [105, 106]. Unlike SARS‐CoV's preferential activation of the EIF2AK3–eIF2A–ATF4–DDIT‐3 axis [107], SARS‐CoV‐2 hijacks the IRE1–XBP1 pathway to remodel the ER, upregulating NUAK2 to enhance receptor stabilization and viral internalization [108]. The nonstructural protein 6 (NSP6) further amplifies ERS, activating EIF2AK3–eIF2A‐mediated autophagy [109]. In infected epithelial cells, IRE1/EIF2AK3‐driven ERS not only causes surfactant deficiency and cellular reprogramming but also triggers EIF2AK3–*ATF4*–DDIT‐3‐dependent mitochondrial apoptosis and NF‐κB‐mediated cytokine production, collectively contributing to epithelial dysfunction and lung injury [110, 111].

#### Monocytes and macrophages

SARS‐CoV‐2 exploits ERS to enhance infectivity and immune evasion in a cell‐type‐specific manner. In myeloid cells, elevated BiP/HSPA5 not only mediates viral entry in ACE2‐deficient monocytes but also sustains UPR activation in macrophages [112, 113]. The viral accessory proteins open reading frame 3a (ORF3a) and open reading frame 8 (ORF8) further manipulate ERS through divergent mechanisms: ORF3a induces protein aggregation and pro‐inflammatory responses that fuel cytokine storms, whereas ORF8 initiates reticulophagy and the calnexin switch to disrupt ER proteostasis and enhance viral replication [114, 115, 116, 117, 118]. These ERS‐mediated effects collectively contribute to COVID‐19 pathogenesis by upregulating surface BiP on leukocytes to amplify hyperinflammation in acute respiratory distress syndrome (ARDS) [119], and by enabling the envelope protein to dynamically regulate NLRP3 inflammasome activation in macrophages [120].

### Tuberculosis (TB)

TB, caused by *Mycobacterium tuberculosis* (Mtb), hijacks ERS to subvert host immunity. While ERS mediates host defense via granuloma formation, Mtb subverts it to block phagolysosome maturation and manipulate apoptosis/autophagy through virulence factors such as B‐cell lymphoma 2‐associated athanogene 2 (BAG2) and cadherin‐15 (CDH15).

#### Macrophages

ERS orchestrates a multifaceted battle between host defense and Mtb pathogenesis through the following macrophage pathways: (1) the host‐protective axis in which ERS activates the EIF2AK3–eIF2A–ATF4 cascade to drive M1 polarization, augmenting autophagy and apoptosis to eliminate intracellular Mtb [121, 122], while simultaneously enhancing autophagic flux via BAG2‐mediated mechanisms – including extracellular signal‐regulated kinase (ERK)‐dependent Beclin‐1/B‐cell lymphoma 2 (BCL2) dissociation and XBP1‐mediated SQSTM1/p62 recruitment to ER membranes for reticulophagy [123]; (2) Mtb's early immune evasion strategy employing virulence factors like the PE_PGRS1 that specifically downregulate EIF2AK3 phosphorylation and Rv3033 that blocks both mitochondrial cytochrome c release and IRE1‐dependent ERS signaling, thereby inhibiting apoptosis while promoting M2 polarization [121, 124, 125]; and (3) late‐stage pathogenic dissemination where Mtb switches to pro‐apoptotic tactics through Rv0297's TLR4‐dependent ERS induction, CDH15‐induced ER structural damage, and Rv1515c‐mediated cytokine modulation [126, 127, 128]. Parallel to these processes, ERS activates two distinct inflammasome pathways to drive interleukin‐1 beta (IL‐1β) production: the classical NLRP3‐caspase‐1 pyroptosis pathway and an alternative pathway dependent on caspase‐8, mitochondrial ROS, and BH3‐interacting domain death agonist (Bid) [129, 130].

#### Monocytes

During Mtb infection, ERS orchestrates divergent monocyte responses through distinct pathways: whereas Ca^2+^‐ROS‐inhibitor of IkappaB kinase (IKK2)‐mediated ERS promotes pathological DDIT‐3‐dependent apoptosis and pro‐inflammatory cytokine production [131], granzyme A induces a protective ERS program that upregulates ATP generation and downregulates ferroportin‐1 (FPN1) via DDIT‐3, creating an iron‐restricted microenvironment that inhibits intracellular mycobacterial growth while maintaining monocyte viability [132].

## Intercellular crosstalk: integrating ERS across cellular networks to shape lung pathology

While cell‐type‐specific ERS responses constitute a fundamental layer of pulmonary disease mechanisms, the integrated pathogenesis emerges from complex, maladaptive intercellular dialogues that are initiated and amplified by ERS. Functioning as a central signaling hub, ERS reprograms the secretory profile of stressed cells, thereby broadcasting molecular signals that orchestrate the behavior of neighboring cells. This cell‐to‐cell communication establishes a self‐reinforcing pathological interactome that drives and sustains tissue‐level dysfunction.

For example, during fibrotic remodeling, ERS in alveolar epithelial cells promotes fibroblast activation through DDIT‐3‐dependent paracrine signaling, including mediators such as Sonic hedgehog [133]. Conversely, cigarette smoke‐induced ERS in lung fibroblasts impairs Wnt/β‐catenin‐mediated epithelial–mesenchymal crosstalk, compromising alveolar repair and contributing to the pathophysiology of COPD [134], collectively illustrating a bidirectional breakdown in epithelial–stromal communication.

Furthermore, ERS disrupts immune‐structural interactions, as evidenced in acute lung injury where ERS in macrophages exacerbates epithelial damage via TRAIL secretion [135]. Similarly, under endoplasmic reticulum and oxidative stress, lung epithelial‐derived IL‐1α acts as a pivotal alarmin which – through TLR3/NF‐κB‐mediated signaling – triggers a robust pro‐inflammatory phenotype in lung fibroblasts, thereby driving neutrophilic inflammation and tissue remodeling during exacerbations of chronic lung diseases [136].

Notably, ERS‐mediated intercellular communication can extend beyond pulmonary boundaries, as demonstrated by the ability of pulmonary epithelial ERS to generate extracellular vesicles that systemically activate brain endothelial cells [137]. Together, these observations reposition the UPR not merely as a cell‐autonomous adaptive mechanism but as a master regulator of pathological cellular networks that ultimately determines the phenotypic expression of lung disease.

## Limitations and critical assessment of current research

While significant advances have been made in elucidating the role of ERS and UPR in lung diseases, a critical appraisal of the current literature reveals several persistent challenges that must be addressed to move the field forward. The translational potential of these findings is currently constrained by limitations in model systems, mechanistic resolution, and clinical applicability.

A primary challenge lies in the existing methodological frameworks. The field remains heavily dependent on *in vitro* systems and preclinical animal models, which, while invaluable, only partially recapitulate the chronicity and cellular complexity of human diseases such as idiopathic pulmonary fibrosis (IPF) and COPD. Furthermore, the use of potent, acute ER stress inducers, though experimentally convenient, may not accurately model the subtle, persistent proteostatic stress observed in human pathology. The interpretation of studies using global UPR knockout models is also often complicated by developmental compensations.

We also identified significant gaps in our mechanistic understanding. A major hurdle is the lack of tools to visualize and quantify UPR activation with spatiotemporal and cell‐type‐specific resolution *in vivo*. This gap impedes a nuanced understanding of how individual UPR branches differentially drive disease pathogenesis over time. Many clinical correlations, while suggestive, still lack robust causal validation in human tissues. Moreover, the crosstalk between ERS and other key pathways – such as mitochondrial quality control and immunometabolism – is often studied in isolation, leaving an integrated view of the pathological network underdeveloped.

Finally, the path to clinical translation faces considerable hurdles. Although numerous UPR‐modulating compounds show promise in preclinical studies (supplementary material, Table S2), their branch selectivity, safety profiles, and efficacy in humans remain largely unexplored. The absence of validated biomarkers for UPR activation in patient cohorts further hinders the design of targeted clinical trials.

## Future perspectives

To translate mechanistic insights into viable therapies, future research must address several translational gaps. A key priority is to delineate the precise spatiotemporal dynamics of UPR activation across different lung cell types *in vivo*. Emerging approaches – such as single‐cell multi‐omics and genetically encoded UPR biosensors – will help to clarify which UPR branches are activated in which cells and at what stage of disease, thereby identifying actionable therapeutic windows. This high‐resolution mapping will in turn guide the rational design of branch‐selective UPR modulators, shifting the focus from broad‐spectrum ERS agents to compounds that can differentially modulate pathway outputs according to cellular context.

In parallel, translational development requires robust biomarkers that reflect UPR activity in patient samples, enabling better patient stratification. Candidate modulators should be evaluated in human‐relevant models, notably patient‐derived lung organoids, which preserve disease‐specific cellular interactions. The long‐term objective is to integrate such targeted UPR modulators with existing therapies through combination strategies that are informed by a detailed understanding of how UPR signaling is rewired within disease‐specific cellular networks. For reference, a complete list of abbreviations used in this review is provided in supplementary material, Table S3.

## Conclusions

In summary, ERS represents a dynamic and influential pathway in lung health and disease. Its dual role – balancing proteostatic adaptation and cell death – necessitates careful investigation into the timing, context, and branch specificity of UPR activation. Critical assessment of current research highlights the need for more physiologically relevant models, improved mechanistic dissection, and validated biomarkers to bridge the gap between preclinical discovery and clinical application. Moving forward, leveraging advanced technologies and accelerating the translational evaluation of UPR‐targeting agents will be crucial for developing effective therapeutic strategies for patients with progressive lung diseases.

## Author contributions statement

LLS was responsible for investigation, data curation, formal analysis, conceptualization, methodology and writing – original draft. YCL provided resources, supervision, and contributed to writing – review and editing. CX conducted investigation and contributed to writing – review and editing. YCZ and KMX performed investigation. DY and XYH were responsible for funding acquisition and contributed to writing – review and editing.

## Supporting information


**Figure S1.** ERS in lung diseases: cell‐type‐specific mechanisms of pathogenesis
**Table S1**. ERS biomarkers and their correlation with lung disease severity and prognosis
**Table S2**. ERS‐targeting inhibitors and their therapeutic applications in lung diseases
**Table S3**. Abbreviations and full terms used in this study

## Data Availability

Data sharing is not applicable to this article as no new data were created or analyzed.
