# Supplementary material for: Endoplasmic reticulum stress and the unfolded protein response in lung diseases: molecular pathways and therapeutic interventions
Source: J Pathol. 2026 Apr 14;269(3):268–83. doi: 10.1002/path.70058 (PMC13238395; doi:10.1002/path.70058)
Supplement: Supplementary file 1 — Figure S1. ERS in lung diseases: cell‐type‐specific mechanisms of pathogenesis Table S1. ERS biomarkers and their correlation with lung disease severity and prognosis Table S2. ERS‐targeting inhibitors and their therapeutic applications in lung diseases Table S3. Abbreviations and full terms used in this study [file PATH-269-268-s001.docx]

**Endoplasmic reticulum stress and the unfolded protein response in lung diseases: molecular pathways and therapeutic interventions**

L Song, Y Liu, *et al. J Pathol* <https://doi.org/10.1002/path.70058>

**Supplementary Figure S1**

**Supplementary Tables S1–S3**

Reference numbers refer to the main text list

**
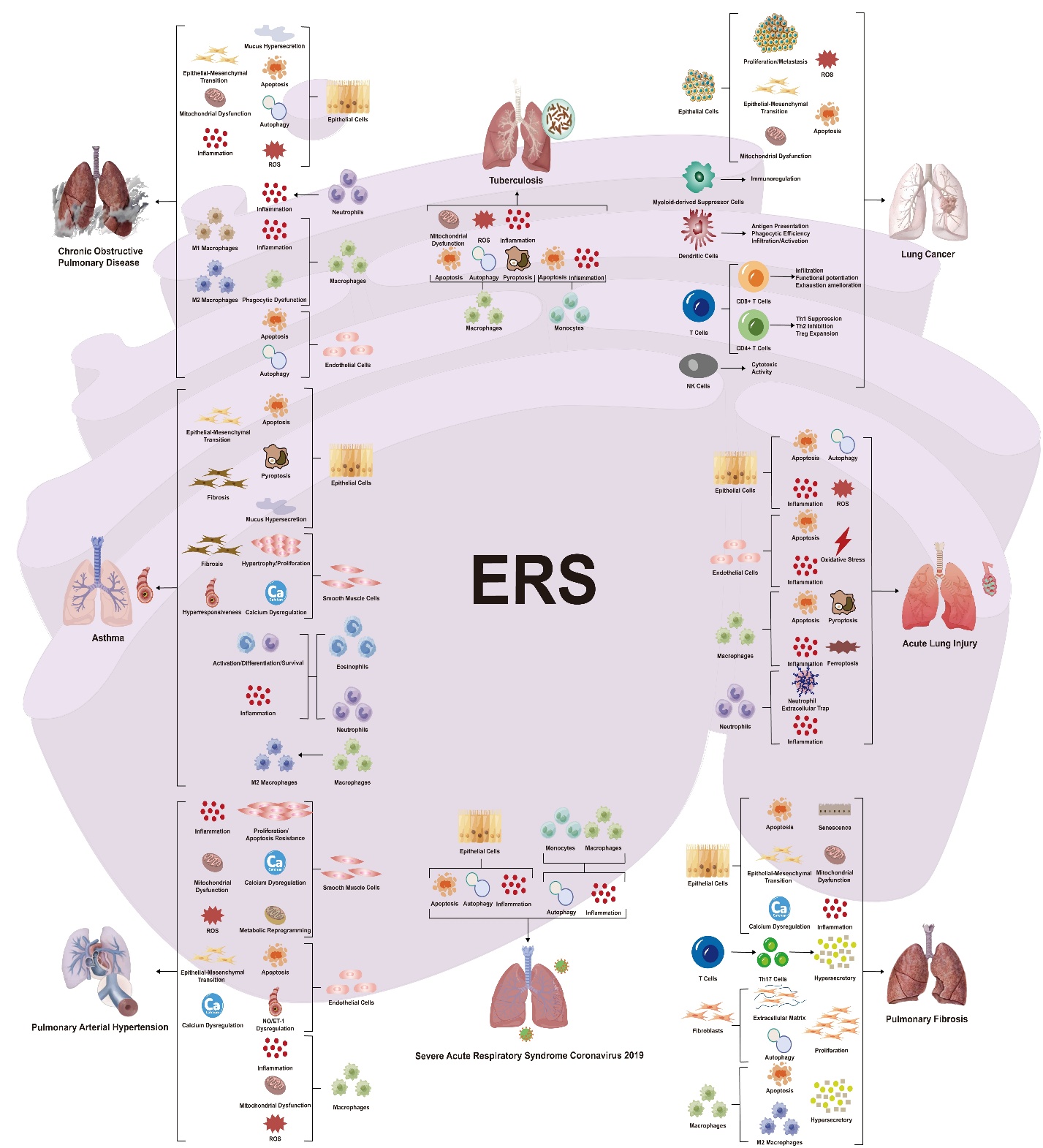
**

**Figure S1. ERS in lung diseases: cell-type-specific mechanisms of pathogenesis.** Diagram illustrating how ERS modulates different lung cell types to promote disease progression, with emphasis on key pathological processes including inflammation, apoptosis, and fibrosis.

**Table S1.** ERS biomarkers and their correlation with lung diseases severity and prognosis.

| **Biomarker** | **Associated disease** | **Clinical significance** | **Refs** |
| --- | --- | --- | --- |
| XBP1 | **COPD** | Predicts rapid FEV1 decline in COPD and at-risk smokers | [138] |
| BiP/HSPA5 |  | Detects lung damage and early COPD | [139] |
| BiP/HSPA5, XBP1s | Lung cancer | Predicts treatment response and prognosis | [140–142] |
| Calreticulin |  | Prognostic biomarker and therapeutic target | [29] |
| GRP94 |  | Prognostic biomarker and therapeutic target | [34] |
| EIF2AK3, IRE1 | Asthma | Predicts severe asthma | [143] |
| BiP/HSPA5 | PAH | Risk stratification biomarker | [144] |
| BiP/HSPA5, XBP1, DDIT-3, EIF2AK3, eIF2A | PF | Diagnostic and prognostic indicators | [145,146] |
| BiP/HSPA5 | SARS-CoV-2 infection | Predicts severe pneumonia and prognosis | [119,147] |
| ATF4, HSPA5, DDIT-3 | TB | Correlated with disease severity and complications | [148,149] |

ERS, endoplasmic reticulum stress; XBP1, X-box binding protein 1; BiP, binding immunoglobulin protein; HSPA5, endoplasmic reticulum chaperone BiP; COPD, chronic obstructive pulmonary disease; FEV1, forced expiratory volume in 1 second; XBP1s, spliced X-box binding protein 1; GRP94, endoplasmin; EIF2AK3, eukaryotic translocation initiation factor 2-alpha kinase 3; IRE1, serine/threonine-protein kinase/endoribonuclease; PAH, pulmonary arterial hypertension; DDIT-3, DNA damage-inducible transcript 3 protein; eIF2A, eukaryotic initiation factor 2A; PF, pulmonary fibrosis; SARS-CoV-2, severe acute respiratory syndrome coronavirus 2; ATF4, activating transcription factor 4; TB, tuberculosis.

**Table S2.** ERS-targeting inhibitors and their therapeutic applications in lung diseases.

| **ERS-targeting inhibitors** | **Potential compounds** | **Preclinical model** | **Associated disease** | **Significance** | **Refs** |
| --- | --- | --- | --- | --- | --- |
| **UPR pathway modulator inhibition** | | | | | |
| IRE1/XBP1 | 4µ8c | *In vitro* | Asthma | Suppresses IL-5 production in Th2 cells by inhibiting IRE1, mitigating acute exacerbations | [150] |
|  |  | *In vivo*/*in vitro* | ALI | Attenuates LPS-induced endoplasmic reticulum stress (ERS) via IRE1–XBP1 axis blockade, downregulating TXNIP/NLRP3 inflammasome and ERK/NF-κB signaling | [151] |
|  |  | *In vivo*/*in vitro* | PF | Synergizes with CSP to inhibit collagen secretion and enhance MMP-mediated ECM degradation in TGFβ-treated fibroblasts | [152] |
|  | MKC8866 | *In vitro* | PF | Abrogates TGF-β1-driven XBP1 splicing, suppressing COL1A2/fibronectin overproduction | [95] |
|  |  | *In vivo* | SARS-CoV-2 infection | Blocks viral ERS and cytokine storms by targeting TLR8/IRE1 signaling in dendritic cells | [153] |
|  | STF-083010 | *In vivo*/*in vitro* | ALI | Reduces BiP and XBP-1s levels and restores β-catenin homeostasis in airway epithelium | [58] |
|  | KIRA | *In vivo* | PF | Attenuates IRE1/XBP1/ATF4 signaling and reduces ECM deposition in bleomycin-induced fibrosis | [154] |
| EIF2AK3/eIF2A | GSK2656157 | *In vitro* | PF | Reverses fibrosis by normalizing macrophage FAO via EIF2AK3–ATF4–PPARGC1α axis inhibition | [155] |
|  |  | *In vitro* | TB | Reduces BCG-induced pyroptosis and pulmonary pathology | [17] |
|  |  | *In vitro* | SARS-CoV-2 infection | Attenuates apoptosis and lung damage | [156] |
|  | GSK2606414 | *In vitro*/*in vivo* | ALI | Improves alveolar–capillary barrier integrity and reduces neutrophil infiltration | [53] |
|  |  | *In vitro* | SARS-CoV-2 infection | Dual inhibition of mitochondrial apoptosis and NF-κB-driven inflammation | [111] |
|  | NCI 159456 | *In vitro* | Lung cancer | Selectively induces apoptosis via ATF4/DDIT3/BAX upregulation and BCL-2 suppression, sparing normal fibroblasts | [157] |
|  | Salubrinal | *In vitro* | COPD | Preserves EIF2AK3–eIF2A homeostasis, protecting bronchial epithelia from CSE-induced apoptosis | [158] |
|  |  | *In vitro* | ALI | Prevents eIF2A dephosphorylation and DDIT-3 upregulation in lung epithelial cells | [159] |
|  |  | *In vitro* | PF | Synergizes with 3-methyladenine to block SiO₂-induced fibroblast activation via dual inhibition of PPP1R13B-mediated ERS and autophagic flux, suppressing proliferation/migration | [160] |
|  | ISRIB | *In vivo* | PF | Ameliorates lung fibrosis in murine models by reducing profibrotic macrophage recruitment and restoring eIF2B-mediated protein translation, thereby suppressing ATF4 target gene expression | [161] |
|  |  | *In vivo* | TB | Enhances TB treatment efficacy in murine models by accelerating bacterial clearance and improving lung pathology via suppression of hyperactivated ISR pathways as adjunctive therapy | [162] |
| ATF6 | Melatonin | *In vivo* | COPD | Downregulates HSPA5/DDIT-3 and NLRP3 inflammasome, preserving alveolar architecture | [163] |
|  |  | *In vitro*/*in vivo* | PF | Reduces pro-inflammatory cytokines in smoke/LPC-exposed models and attenuates fibrotic signaling in lung tissues | [164] |
|  |  | *In vitro*/*in vivo* | PAH | Disrupts MIF/STAT3/ATF6/autophagy axis, inhibiting PASMC proliferation and vascular remodeling | [165] |
|  | Kaempferol | *In vitro* | Asthma | Dual blockade of IRE1–XBP1 and ATF6 pathways, reducing MUC5AC hypersecretion and goblet cell hyperplasia | [166] |
| **Chemical chaperone** | | | | | |
| Non-specific | 4-PBA | *In vitro*/*in vivo* | COPD | Inhibits cigarette smoke-induced ERS, suppresses NF-κB signaling, and reduces alveolar cell apoptosis | [167] |
|  |  | *In vitro* | Lung cancer | Mitigates NiCl₂-induced ERS in A549 cells by downregulating UPR markers | [16] |
|  |  | *In vivo* | Asthma | Alleviates ovalbumin-induced asthma by suppressing endoplasmic reticulum stress and reducing inflammatory cytokines | [168] |
|  |  | *In vivo*/*in vitro* | ALI | Suppresses ERS markers, inhibiting autophagy and reducing inflammation | [169] |
|  |  | *In vivo*/*in vitro* | PAH | Ameliorates hypoxia-induced pulmonary arterial hypertension by concurrently suppressing all three UPR branches and reducing JNK phosphorylation | [170] |
|  |  | *In vitro* | PF | Reverses hypoxia-triggered alveolar EMT via endoplasmic reticulum stress and HIF signaling | [171] |
|  |  | *In vitro* | SARS-CoV-2 infection | Reduces cs-BiP expression on leukocytes, ameliorating ARDS hyperinflammation. | [119] |
|  |  | *In vitro* | TB | Modulates mitochondrial fission to enhance mycobacterial clearance | [172] |
|  | TUDCA | *In vivo* | Asthma | Suppresses allergic asthma responses by reducing Th2 cytokines, inhibiting mucus metaplasia, and modulating UPR transducers in murine models | [173] |
|  |  | *In vivo* | ALI | Inhibits LPS-induced lung injury by suppressing endoplasmic reticulum stress markers, reducing cytokines, and preserving vascular integrity | [174] |
|  |  | *In vitro*/*in vivo* | PF | Attenuates pulmonary fibrosis by downregulating HSPA5 expression while reducing hSP9α secretion and collagen I deposition | [175] |
|  |  | *In vitro* | Influenza A viral infection | Selectively inhibits IRE1-dependent viral replication without affecting the EIF2AK3–ATF6 pathway | [176] |
| **Chaperone regulator inhibition** | | | | | |
| HSP70 | VER-155008 | *In vitro* | Lung cancer | Synergizes with autophagy inhibitors to potentiate cisplatin-induced apoptosis via Hsp70–AMPK axis disruption | [177] |
| HSP90 | PU-H71 | *In vitro* | Lung cancer | Enhances the radiosensitivity of lung cancer cells by inhibiting DNA double-strand break repair | [178] |
| HSPA5 | YUM70 | *In vitro* | SARS-CoV-2 infection | Targets HSPA5 to inhibit viral entry and lung damage in murine models | [179] |
| **ER-associated degradation enhancer** | | | | | |
| p97/VCP | Eeyarestatin I | *In vitro*/*in vivo* | Lung cancer | Induces G0/G1 arrest and apoptosis by blocking p97/VCP-dependent ERAD | [180] |
| USP14/UCHL5 | b-AP15 | *In vitro*/*in vivo* | Lung cancer | Promotes EGFR ubiquitination and degradation, suppressing tumor growth | [181] |

ERS, endoplasmic reticulum stress; IRE1, serine/threonine-protein kinase/endoribonuclease; XBP1, X-box binding protein 1; IL-5, interleukin 5; Th2, T helper 2; ALI, acute lung injury; LPS, lipopolysaccharide; TXNIP, thioredoxin-interacting protein; NLRP3, NLR family pyrin domain containing 3; ERK, extracellular signal-regulated kinase; NF-κB, NF-kappa-B inhibitor-interacting Ras-like protein; PF, pulmonary fibrosis; CSP, connective tissue growth factor secretion pathway; MMP, matrix metalloproteinase; ECM, **extracellular matrix; TGFβ, transforming growth factor beta;** TNF-α, **tumor necrosis factor alpha; IL-6,** interleukin 6; TGF-β1, transforming growth factor-beta 1; COL1A2, collagen type 1 alpha 2; SARS-CoV-2, severe acute respiratory syndrome coronavirus 2; TLR8, Toll-like receptor 8; BiP, binding immunoglobulin protein; XBP1s, spliced X-box binding protein 1; ATF4, activating transcription factor 4; FAO, fatty acid oxidation; **PPARGC1α,** peroxisome proliferator-activated receptor gamma coactivator 1-alpha; TB, tuberculosis; BCG, Bacillus Calmette-Guérin; COPD, chronic obstructive pulmonary disease; CSE, cigarette smoke extract; DDIT-3, DNA damage-inducible transcript 3 protein; PPP1R13B, protein phosphatase 1 regulatory subunit 13B; eIF2B, eukaryotic initiation factor 2B; ISRIB, integrated stress response inhibitor; LPC, **lysophosphatidylcholine;** PAH, pulmonary arterial hypertension; MIF, macrophage migration inhibitory factor; STAT3, **signal transducer and activator of transcription 3;** ATF6, **activating transcription factor 6; PASMC, pulmonary artery smooth muscle cell; MUC5AC, mucin 5AC;** p-eIF2A, phosphorylated eukaryotic initiation factor 2 alpha; **JNK, stress-activated protein kinase;** EMT, epithelial–mesenchymal transition; HIF, hypoxia-inducible factor; cs-BiP, cell surface binding immunoglobulin protein; ARDS, acute respiratory distress syndrome; hSP9α, human splicing factor proline/glutamine-rich 9 alpha; MPO, myeloperoxidase; Hsp70, heat shock protein 70; AMPK, AMP-activated protein kinase; ERAD, endoplasmic reticulum-associated degradation; EGFR, epidermal growth factor receptor.

**Table S3** Abbreviations and full terms used in this study.

| **Abbreviation** | **Full name** |
| --- | --- |
| ACE2 | Angiotensin-converting enzyme 2 |
| AECII | Alveolar epithelial type II cells |
| AKT | AKT serine/threonine kinase |
| ALI | Acute lung injury |
| AMs | Alveolar macrophages |
| ANG1–7 | Angiotensin-(1–7) |
| ANGII | Angiotensin II |
| α-SMA | Alpha smooth muscle actin |
| ATF4 | Activating transcription factor 4 |
| ATF6 | Activating transcription factor 6 |
| BAG2 | B-cell lymphoma 2-associated athanogene 2 |
| BCL-2 | B-cell lymphoma 2 |
| Bid | BH3-interacting domain death agonist |
| BiP | Binding immunoglobulin protein |
| CD47 | Cluster of differentiation 47 |
| CD91 | Cluster of differentiation 91 |
| CDH15 | Cadherin-15 |
| cGAS | Cyclic GMP-AMP synthase |
| DDIT-3 | DNA damage-inducible transcript 3 protein |
| COPII | Coat protein complex II |
| COPD | Chronic obstructive pulmonary disease |
| CS | Cigarette smoke |
| DCs | Dendritic cells |
| eIF2A | Eukaryotic initiation factor 2A |
| eIF2B | Eukaryotic initiation factor 2B |
| EMT | Epithelial–mesenchymal transition |
| ER | Endoplasmic reticulum |
| ERAD | ER-associated degradation |
| ERK | Extracellular signal-regulated kinase |
| ERO1α/β | ERO1-like protein alpha/beta |
| PDIA3 | Protein disulfide-isomerase A3 |
| ERS | Endoplasmic reticulum stress |
| ET-1 | Endothelin-1 |
| EVs | Extracellular vesicles |
| FADK 1 | Focal adhesion kinase 1 |
| GNAQ/GNA11 | Guanine nucleotide-binding protein G(q) subunit alpha/guanine nucleotide-binding protein subunit alpha-11 |
| GRP94 | Endoplasmin |
| HDM | House dust mite |
| HSP70 | Heat shock protein 70 |
| HSP90 | Heat shock protein 90 |
| HSPA5 | Endoplasmic reticulum chaperone BiP |
| IFN-γ | Interferon-gamma |
| IKK2 | IkappaB kinase |
| IL-1β | Interleukin-1 beta |
| IL-4 | Interleukin 4 |
| IL-4Rα | IL-4 receptor subunit alpha |
| IL-10 | Interleukin 10 |
| IL-17 | Interleukin 17 |
| IRE1 | Serine/threonine-protein kinase/endoribonuclease |
| IRF4 | Interferon regulatory factor 4 |
| ISRIB | Integrated stress response inhibitor |
| JNK | Stress-activated protein kinase |
| M1 | Classically activated macrophage |
| M2 | Alternatively activated macrophage |
| MAPK | Mitogen-activated protein kinase |
| MERS-CoV | Middle East respiratory syndrome coronavirus |
| MKK5 | Mitogen-activated protein kinase kinase 5 |
| Mtb | *Mycobacterium tuberculosis* |
| mTOR | Mechanistic target of rapamycin |
| MUC5AC | Mucin 5AC |
| NET | Neutrophil extracellular trap |
| NETosis | Neutrophil extracellular trap formation |
| NF-κB | NF-kappa-B inhibitor-interacting Ras-like protein |
| NLRP3 | NLR family pyrin domain containing 3 |
| NO | Nitric oxide |
| RTN4 | Reticulon-4 isoform B |
| NRF1 | Nuclear respiratory factor 1 |
| NRF2 | Nuclear factor erythroid 2-related factor 2 |
| NUAK2 | NUAK family kinase 2 |
| ORF | Open reading frame |
| ORF3a | Open reading frame 3a |
| ORF8 | Open reading frame 8 |
| ORMDL3 | Orosomucoid-like protein 3 |
| 4-PBA | 4-Phenylbutyrate |
| PAECs | Pulmonary artery endothelial cells |
| PAH | Pulmonary arterial hypertension |
| PASMCs | Pulmonary artery smooth muscle cells |
| PDI | Protein disulfide isomerase |
| p-eIF2A | Phosphorylated eukaryotic initiation factor 2A |
| EIF2AK3 | Eukaryotic translocation initiation factor 2-alpha kinase 3 |
| PF | Pulmonary fibrosis |
| PI3K-δ | Phosphoinositide 3-kinase delta |
| p-JNK | Phosphorylated stress-activated protein kinase |
| PPARγ | Peroxisome proliferator-activated receptor gamma |
| RAE1 | Retinoic acid early transcript 1 |
| RhoA | Ras homolog family member A |
| RIDD | Regulated IRE1-dependent decay |
| ROCK | Rho-associated protein kinase |
| ROS | Reactive oxygen species |
| S1P | Site-1 protease |
| S2P | Site-2 protease |
| SARS-CoV-2 | Severe acute respiratory syndrome coronavirus 2 |
| SFTPC | Surfactant protein C |
| SMAD2/3 | SMAD family member 2/3 |
| SQSTM1/p62 | Sequestosome-1 |
| STAT6 | Signal transducer and activator of transcription 6 |
| STING | Stimulator of interferon genes |
| TB | Tuberculosis |
| TFEC | Transcription factor EC |
| TGF-β1 | Transforming growth factor beta 1 |
| TGFBR1 | Transforming growth factor beta receptor 1 |
| Th1 | T helper 1 |
| Th17 | T helper 17 |
| Th2 | T helper 2 |
| TLR4 | Toll-like receptor 4 |
| TMAO | Trimethylamine N-oxide |
| TNF | Tumor necrosis factor |
| TRAF2 | TNF receptor-associated factor 2 |
| TRAIL-R | TNF-related apoptosis-inducing ligand receptor |
| Treg | Regulatory T cell |
| TUDCA | Tauroursodeoxycholic acid |
| TXNDC5 | Thioredoxin domain-containing protein 5 |
| UPR | Unfolded protein response |
| XBP1 | X-box binding protein 1 |
| XBP1s | Spliced X-box binding protein 1 |
